# Recombinant Cyclodextrinase from *Thermococcus kodakarensis* KOD1: Expression, Purification, and Enzymatic Characterization

**DOI:** 10.1155/2015/397924

**Published:** 2015-01-26

**Authors:** Ying Sun, Xiaomin Lv, Zhengqun Li, Jiaqiang Wang, Baolei Jia, Jinliang Liu

**Affiliations:** ^1^College of Plant Sciences, Jilin University, Changchun 130062, China; ^2^Department of Life Science, Chung-Ang University, Seoul 156-756, Republic of Korea

## Abstract

A gene encoding a cyclodextrinase from *Thermococcus kodakarensis* KOD1 (CDase-Tk) was identified and characterized. The gene encodes a protein of 656 amino acid residues with a molecular mass of 76.4 kDa harboring four conserved regions found in all members of the *α*-amylase family. A recombinant form of the enzyme was purified by ion-exchange chromatography, and its catalytic properties were examined. The enzyme was active in a broad range of pH conditions (pHs 4.0–10.0), with an optimal pH of 7.5 and a temperature optimum of 65°C. The purified enzyme preferred to hydrolyze *β*-cyclodextrin (CD) but not *α*- or *γ*-CD, soluble starch, or pullulan. The final product from *β*-CD was glucose. The *V*_max_ and *K*_*m*_ values were 3.13 ± 0.47 U mg^−1^ and 2.94 ± 0.16 mg mL^−1^ for *β*-CD. The unique characteristics of CDase-Tk with a low catalytic temperature and substrate specificity are discussed, and the starch utilization pathway in a broad range of temperatures is also proposed.

## 1. Introduction

Cyclodextrins (CDs) are cyclic maltooligosaccharides of at least 5 or more *α*-D-glucopyranoside units linked via *α*(1,4)-glycosidic bonds such as that found in amylose (a fragment of starch). The most common CDs are *α*-, *β*-, and *γ*-CDs, which have 6, 7, or 8 D-glucopyranoside units, respectively [1]. All of the hydroxyl groups in CDs are oriented to the outside of the ring, while the glycosidic oxygen and two rings of nonexchangeable hydrogen atoms are directed toward the interior of the cavity. This combination provides CDs a hydrophobic inner cavity and a hydrophilic exterior [2]. The hydrophobic environment of the cavity enables CDs to form inclusion complexes with many water-insoluble compounds that have numerous useful applications in the food, pharmaceutical, drug delivery, and chemical industries as well as in agriculture and environmental engineering [3]. The increasing application of CDs showing varying degrees of resistance to hydrolysis by common amylases has stimulated interest in the research of CD-degrading enzymes [4]. CD can be obtained from starch by the action of cyclomaltodextrin glucanotransferase (CGTase), which is a member of the *α*-amylase family of glycosyl hydrolases (family 13). CGTases are usually classified into 3 subgroups (*α*-, *β*-, and *γ*-CGTases) according to the different CD specificities of *α*-, *β*-, or *γ*-CD [5]. For example, the CGTase from* Pyrococcus furiosus* is a *β*-CGTase [6]. The first halophilic archaeal CGTase isolated from the halophilic archaeon* Haloferax mediterranei* mainly produces *α*-CD followed by *β*-CD and *γ*-CD with ratios of 1 : 1, 1 : 0.6, and 1 : 0.3, respectively, as determined by spectrophotometric assays [7].

The CD-degrading enzymes, including cyclomaltodextrinase (CDase, EC 3.2.1.54), maltogenic amylase (EC 3.2.1.133), and neopullulanase (EC 3.2.1.135), have been categorized into a common subfamily in glycoside hydrolase family 13 (GH13) and have been reported to be capable of hydrolyzing all or two of the following three types of substrates: CD, pullulan, and starch [1]. CDase is a unique enzyme that catalyzes the hydrolysis of CDs much faster than pullulan and starch to form linear oligosaccharides of *α*-1,4-linkages, and it can release substances from CD inclusion complexes [1]. Since the CDase from* Bacillus macerans* was first reported in 1968, many studies have been performed with CDases from various bacterial and archaeal sources. Many CDases from bacteria have been characterized, such as enzymes from* Bacillus* [1],* Thermoanaerobacter ethanolicus* strain 39E [8],* Flavobacterium* sp. [9], and* Klebsiella oxytoca* strain M5a1 [10]. Archaea CDases have been characterized from* Archaeoglobus fulgidus* [11],* Thermococcus* sp. B1001 [12],* Thermococcus* sp. CL1 [13],* Thermofilum pendens* [14], and* Pyrococcus furiosus *[15]. Among these CDases, the structure of the CDase from* Flavobacterium* sp. was characterized in detail [16]. This structure suggested that Arg464 functions as a chaperone guiding substrates from solvent into the active center, and Glu340 starts hydrolysis to open the ring [16]. Due to their thermophilic characteristics, CDases from archaea have attracted research interest and have great potential for industrial applications.

*Thermococcus kodakarensis* KOD1 is a thermophilic anaerobic archaeon whose whole-genome sequence has been reported [17]. As a hyperthermophilic anaerobe living in deep-vent environments,* T. kodakarensis* KOD1 is a model microorganism for studying hyperthermophiles, and it is a potential industrial enzyme source.* T. kodakarensis* KOD1 produces a CGTase (Tk2172) that can predominantly catalyze the formation of *β*-CD [18]. Here, we reported the purification and catalytic characterization of CDase from* T. kodakarensis* KOD1 (CDase-Tk; Tk1770), which hydrolyzes *β*-CD. Together with polysaccharide degradation data, a metabolic pathway for polysaccharide utilization in* T. kodakarensis* KOD1 is proposed.

## 2. Materials and Methods

### 2.1. Microorganisms and Media

*T. kodakarensis* KOD1, which was kindly donated by the Japan Collection of Microorganisms, RIKEN BioResource Center, Japan, was used to isolate genomic DNA, and it was cultured in 280* Thermococcus* medium [17].

### 2.2. Cloning CDase-Tk from* T. kodakarensis* KOD1

PCR using* T. kodakarensis* KOD1 genomic DNA as a template was performed to isolate CDase-Tk using the following oligonucleotide primers: forward: 5′-G GAATTC ATGTATAAGGTTTTCGGG-3′ and reverse: 5′-CCG CTCGAG CTATTCCTGCAGGTCTG-3′ (the underlined bases indicate the restriction enzymes (*Eco*RI and* Xho*I) site). The PCR product and the pET28-(a) vector were digested by the restriction enzymes. The ligation products were transformed into* E. coli* BL21 (DE3) cells by electroporation and confirmed by sequencing.

### 2.3. Expression and Purification of CDase-Tk

*E. coli* BL21(DE3) cells containing the pET28a-CDase-Tk plasmid were cultured in 2 L of LB broth containing 30 *μ*g mL^−1^ kanamycin at 37°C for 3 h. When the OD_600_ reached 0.7, isopropyl-*β*-D-thiogalactopyranoside (IPTG) was added to a final concentration of 1 mM to induce protein expression. After 4 h of culture with shaking, cells were harvested by centrifugation at 6,000 rpm for 10 min at 4°C. The cell pellets were resuspended in lysis buffer (50 mM Tris-HCl, pH 8.0), disrupted by sonication, and centrifuged at 14,000 rpm for 20 min at 4°C. The supernatants were loaded onto a Macro-prep DEAE support column (Amersham Biotech, USA) equilibrated with lysis buffer. Bound proteins were eluted with 50 mM Tris-HCl buffer (pH 8.0) with stepwise increased concentrations of NaCl from 50 to 500 mM. Active CDase-Tk was eluted in the 200 mM NaCl fraction. Protein concentrations were estimated by the Bradford method using bovine serum albumin (BSA) as a standard [19].

### 2.4. Assays for CDase-Tk Activity

CDase-Tk activity was measured by using *α*-, *β*-, and *γ*-CD, soluble starch, and pullulan as substrates. Briefly, an appropriate amount of purified enzyme (approximately 0.80 *μ*g) was added to reaction mixtures (200 *μ*L) containing 0.5% substrate in 20 mM Tris-HCl buffer (pH 7.5), and the reactions were incubated for 1 h at 65°C. The concentrations of reducing sugars liberated from the enzymatic reaction mixture were spectrophotometrically quantified with 3,5-dinitrosalicylic acid reagent at OD = 540 nm in a UV-visible spectrophotometer (Shimadzu, Japan) [20]. One unit of enzyme activity was defined as the amount of enzyme that releases 1 *μ*M of reducing sugar per minute.

The influence of pH on CDase-Tk activity was determined using the protocol described above with the exception of replacing the Tris-HCl buffer with 50 mM sodium acetate (pH 3.0–5.0), 50 mM MES (pH 5.0–7.5), 50 mM HEPES (pH 8.0–8.5), or 50 mM glycine (pH 9.0–10.0) [21]. All assays were performed at the optimal temperature.

For kinetic studies, the initial velocities of enzymatic reactions were examined by varying the concentration of cyclodextrin (from 1 to 10 mg mL^−1^) under optimal conditions. The* Michaelis* constant (*K*_*m*_) value and maximal velocity (*V*_max⁡_) were obtained by mathematical calculations using Sigma Plot (12.5) software. The parameters were determined by three separate experiments.

### 2.5. Thin-Layer Chromatography

Thin-layer chromatography (TLC) of enzymatic hydrolysis products from different substrates was performed with butanol-ethanol-water at a ratio of 4 : 4 : 3 as the mobile phase in silica gel plates. The plates were dipped into a solution containing 0.3% N-(1-naphthyl)-ethylenediamine and 5% H_2_SO_4_ in methanol. Hydrolytic products were visualized by heating the plates at 110°C for 10 min.

## 3. Results and Discussion

### 3.1. Sequence Analysis, Expression and Purification of CDase-Tk

**Figure 1:**
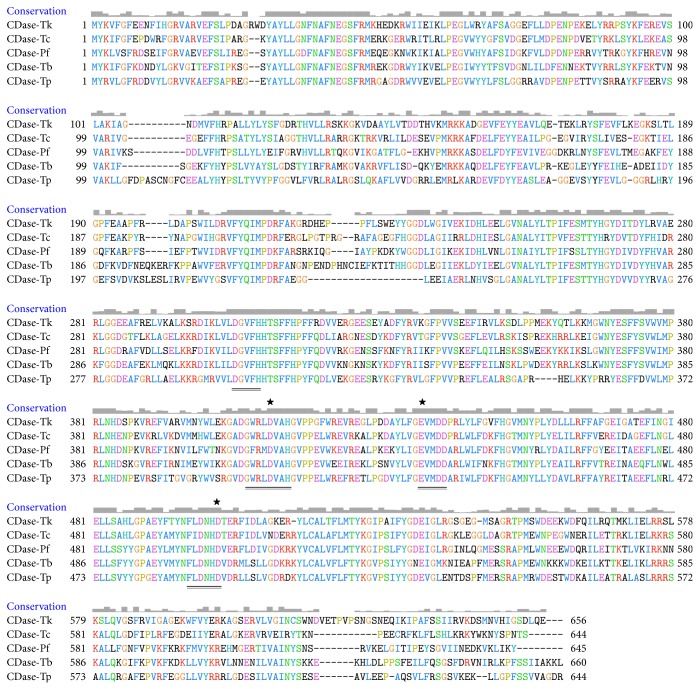
Sequence and structure analysis of CDase-Tk. Cyclodextrinase sequences from* T. kodakarensis* KOD1 (CDase-Tk, Tk1770),* Thermococcus* sp. CL1 (CDase-Tc, YP_006424883.1),* Thermococcus* sp. B1001 (CDase-Tb, BAB18100.1),* Pyrococcus furiosus* (CDase-Pf, NP_579668.1), and* Thermofilum pendens* Hrk 5 (CDase-Tp, YP_920858.1) were aligned. The solid line indicates the four consensus regions conserved in the GH13 family. The asterisks show the positions of the three active sites. The conservation level of each residue is indicated by the height of the bars above each residue. The number at the ending of each line of amino acids indicates the number of the amino acid residues.

CDase-Tk has 656 amino acids, a deduced MW of 76.4 kDa and a pI of 5.5 (http://web.expasy.org/compute_pi/). CDase-Tk does not have a predicted secretion signal. Compared with the CDase sequences available in GenBank, the CDase-Tk sequence is highly similar to that of corresponding genes, for example, genes from strain* Thermococcus* sp. CL1 (59%, YP_006424883.1),* Thermococcus* sp. B1001 (53%, BAB18100.1),* Pyrococcus furiosus* (56%, NP_579668.1), and* Thermofilum pendens* Hrk 5 (52%, YP_920858.1) (Figure 1). A UNIPROTKB Blastp search of the amino acid sequence of CDase-Tk suggested that residues 200–600 contain a signature typical of glycosyl hydrolase (GH) family 13, also known as the *α*-amylase family. The four conserved regions of all GH13 amylolytic enzymes were identified in the CDase-Tk sequence. Figure 1 shows an amino acid sequence alignment of some highly similar GH13 family proteins in which the amino acids Asp411, Glu437, and Asp502 of CDase-Tk correspond to the highly conserved catalytic residues in GH13 CDase.

**Figure 2:**
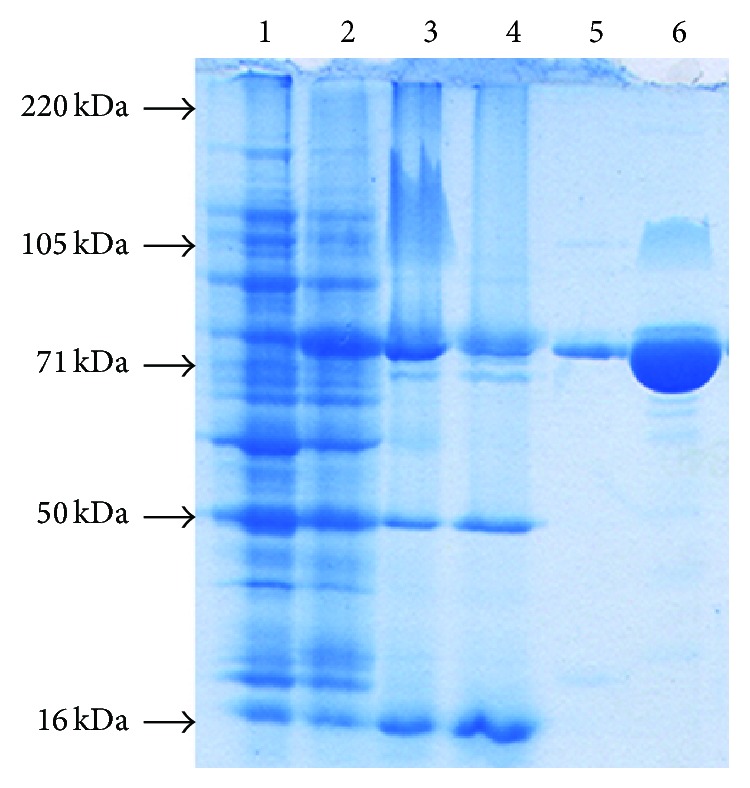
Purification of CDase-Tk. Supernatants of total proteins from recombinant* E. coli* were loaded on a DEAE column, and bound proteins were eluted by stepwise NaCl addition. Molecular mass standards are indicated at the left. Lane 1, crude protein extract from noninduced cells; lane 2, crude protein extract from IPTG-induced cells; lanes 3 and 4, proteins eluted by 50 mM NaCl from the DEAE column; lane 5, proteins eluted by 100 mM NaCl; lane 6, proteins eluted by 200 mM NaCl.

A 1,971-bp fragment of the* CDase-Tk* gene was amplified from genomic DNA from* T. kodakarensis* KOD1 and ligated with the pET28a vector at* Eco*RI and* Xho*I sites to generate the plasmid pET28a-CDase-Tk.* E. coli* cells transformed with pET28a-CDase-Tk were grown and induced to express the gene under the recommended optimal conditions. The enzyme was purified by DEAE column chromatography. The purity and size of isolated proteins were analyzed by SDS-PAGE (Figure 2). CDase-Tk migrates near its predicted molecular weight of ~76 kDa.

### 3.2. Substrate Specificity of CDase-Tk

**Figure 3:**
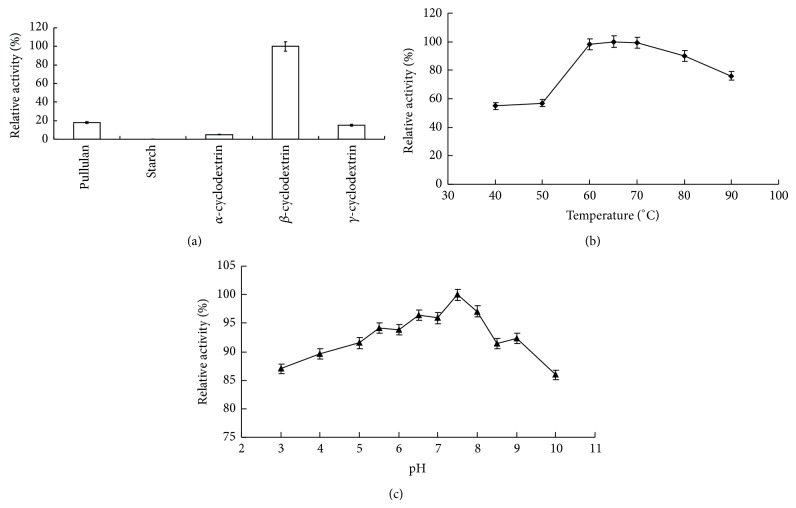
Influence of temperature on the activity and influence of pH on the activity and stability of CDase-Tk. (a) Hydrolytic activity of CDase-Tk to pullulan, starch, and cyclodextrin. (b) Optimal temperature of CDase-Tk. (c) Optimal pH of CDase-Tk. Different buffers were used for the different pH solutions used in this assay. Sodium acetate was used for pHs 3.0, 4.0, and 5.0; MES buffer was used for pHs 5.0 to 7.5; HEPES buffer was used for pHs 8 and 8.5; glycine buffer was used for pHs 9.0 and 10.0. The concentrations of the buffers were 50 mM.

To evaluate the scope of the substrate selectivity of CDase-Tk, five substrates were selected for monitoring of their degradation including *α*-CD, *β*-CD, *γ*-CD, soluble starch, and pullulan.  Figure 3(a) shows the relative activity of the CDs with *β*-CD scaled to 100. CDase-Tk preferred *β*-CD as the most active substrate, and the hydrolyzing activity toward pullulan and *γ*-CD was approximately 20% of that of *β*-CD. Overall, the substrate preference of CDase-Tk is CD ≫ pullulan ≫ starch. This order is somewhat similar to the substrate preference of CDases from other thermophilic archaea, as all of them prefer CD as an optimal substrate (Table 1). However, different thermophilic CDases prefer different CDs. For example, the CDase from* P. furiosus* prefers *α*-CD as a substrate, and the CDase from* T. pendens* prefers to degrade *γ*-CD (Table 1). In addition, the CGTase in* T. kodakarensis* KOD1 predominantly catalyzes the formation of *β*-CD [18], and the substrate specificity of CDase-Tk is in accordance with the CGTase catalytic properties for efficient starch utilization.

### 3.3. pH and Temperature Optima

The recombinant full-length enzyme is active above 30°C, its activity increases together with temperature elevation, and the highest catalytic activity for hydrolyzing *β*-CD could be achieved at 65°C (Figure 3(b)), which is much lower than the optimal growth temperature (85°C) of* T. kodakarensis* KOD1. CDase-Tk showed high similarity in amino acids sequence with CDases from other thermophilic archaea, including* Thermococcus* sp. CL1 (CDase-Tc),* P. furiosus* (CDase-Pf),* Thermococcus* sp. B1001 (CDase-Tb), and* T. pendens* Hrk 5, but the optimal temperature for CDase-Tk is much lower than that for most of these enzymes (approximately 90°C) (Table 1). However, the CGTase in* T. kodakarensis* KOD1 hydrolyzes starch with an optimal temperature of 80°C, which is also lower than the optimal growth temperature for* T. kodakarensis* KOD1 [18].

The pH dependence of CDase-Tk activity was determined using different buffers (50 mM NaAc, pH: 3.0–5.0; 50 mM MES, pH: 5.0–7.5; 50 mM HEPES, pH: 8.0–8.5; and 50 mM glycine, pH: 9.0–10.0). The maximum activity for hydrolyzing *β*-CD was found to be at pH 7.5, which is different from other thermophilic CDases that show their optimal activity at acidic conditions including pHs ranging from 4.5 to 5.5 (Table 1). CDase-Tk was also active at pHs ranging from 4.0 to 10.0 with 89.6, 91.5, 95.9, and 86.0% maximum activity at pHs 4.0, 5.0, 7.0, and 10.0, respectively (set as 100% at pH 7.5) (Figure 3(c)). This result indicates that CDase-Tk can hydrolyze its substrates over a broad pH range, and it should be much suitable for* T. kodakarensis* KOD1 in environmental adaptation.

### 3.4. Kinetic and Product Analysis

**Figure 4:**
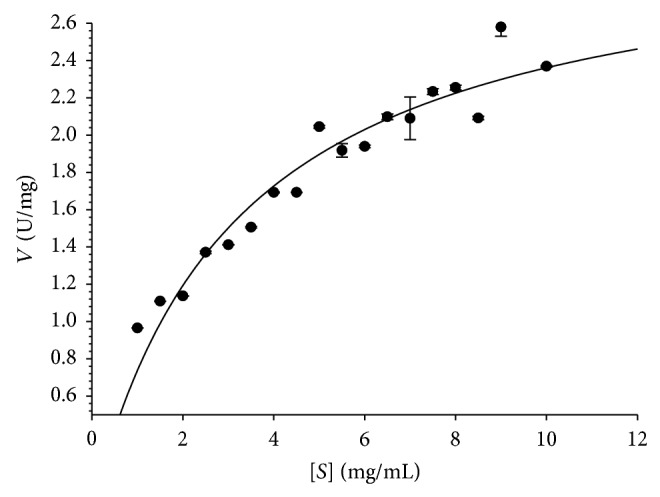
Effects of substrate concentration on the velocity of the cyclodextrinase of CDase-Tk. Assays were performed as described in the Materials and Methods. The parameters reported here are the means of three determinations.

**Figure 5:**
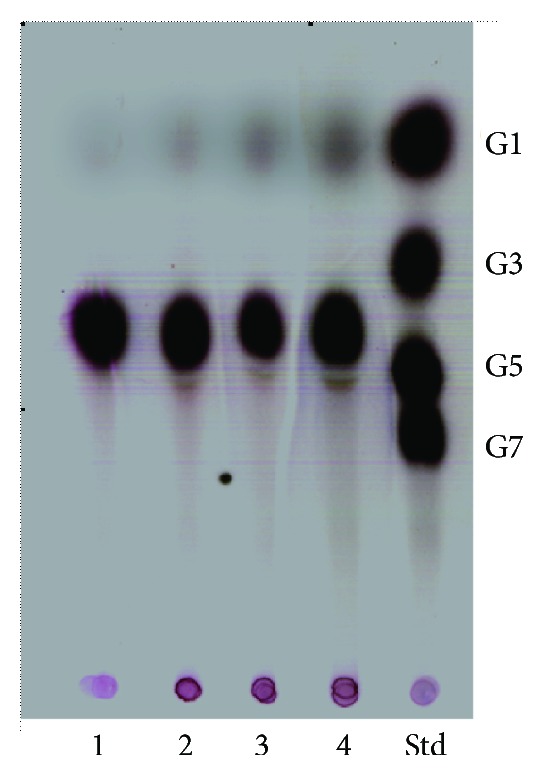
Thin layer chromatography (TLC) of hydrolysis products from *β*-CD generated by CDase-Tk. Lane 1: 1% *β*-CD alone, Lanes 2 to 4: CDase-Tk which was reacted with substrates at 1% concentration at 65°C for 10, 30, or 60 min. Std indicates the oligosaccharide standard containing 1% glucose, maltotriose, maltopentaose, and maltoheptaose.

The kinetics of recombinant CDase-Tk were analyzed using *β*-CD as a substrate by varying its concentration. The reaction was performed in a Tris-HCl buffer (pH 7.5) at 65°C with *β*-CD concentrations ranging from 1 to 10 mg mL^−1^. The Michaelis–Menten equation was used to calculate the kinetic parameters (Figure 4). CDase-Tk catalyzed *β*-CD with *K*_*m*_ = 3.13 ± 0.47 mg mL^−1^ and *V*_max⁡_ = 2.94 ± 0.16 U mg^−1^. TLC results demonstrated that the action of CDase-Tk results in the formation of glucose when using *β*-CD as a substrate (Figure 5). Other CDases show a broad range of substrates and products. For example CDase-Pf, a cyclodextrinase from GH13, possesses characteristics of both *α*-amylase and cyclodextrin-hydrolyzing enzyme. Similar to typical *α*-amylases, CDase-Pf hydrolyzes maltooligosaccharides and starch to mainly produce maltotriose and maltotetraose. However, this enzyme could also attack and degrade pullulan and *β*-CD [15] (Table 1).

## 4. Conclusion

The endocellular cyclodextrinase from the hyperthermophilic archaeon* T. kodakarensis* KOD1 (CDase-Tk) belonging to the GH13 family was heterologously overexpressed in* E. coli* and biochemically characterized. CDase-Tk preferred *β*-CD as its most active substrate, but its activities toward other substrates were hard to measure. In this study, we found that the optimal temperature for enzyme activity is 65°C, and the highest activity was found to be at pH 7.5 with a range of pHs (ranging from 4.0 to 10.0). The characteristic of CDase-Tk hydrolyzing *β*-CD at a relatively low temperature and nonneutral pH should play an important role in the survival of* T. kodakarensis* KOD1 under low temperature conditions (65°C).

**Figure 6:**
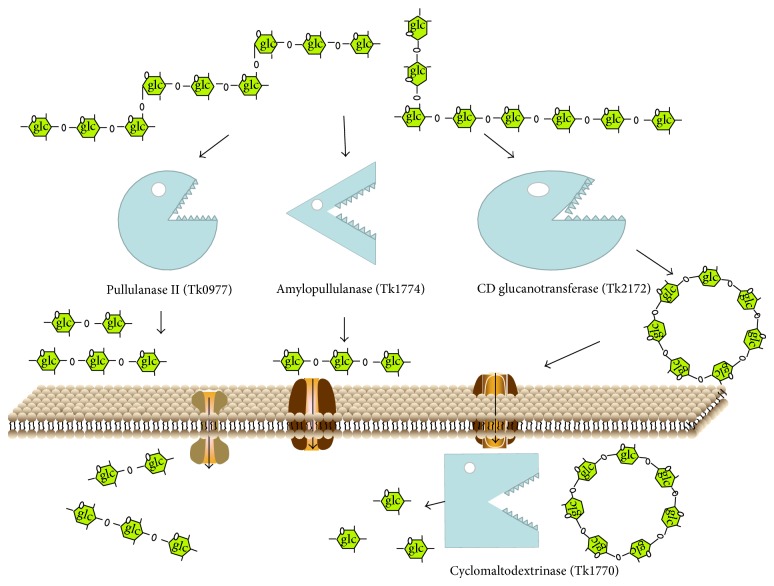
Proposed model for the degradation of starch in* T. kodakarensis* KOD1.

Previously, we reported that two extracellular pullulanases in* T. kodakarensis* KOD1 (Tk0977 and Tk1774) can hydrolyze pullulan and starch to an oligosaccharide with optimal temperatures above 100°C. Tk0977 is a protein of 765 amino acids with a putative 22-residue signal peptide. This protein has four consensus motifs and a catalytic triad of the GH13 family in the deduced amino acid sequence. Tk0977 can effectively hydrolyze starch to produce maltose and maltotriose. Tk1774 is an organic solvent-, detergent-, and thermostable amylopullulanase belonging to the GH57 family of proteins, and it only produces maltotriose [21, 22]. These maltotriose products may be transported by an ABC-type maltodextrin transport system and further enter into the glycolytic pathway. In this study, a pathway comprising a CGTase and CDase in* T. kodakarensis* KOD1 catalyzed the extracellular formation of *β*-CD from starch, and its subsequent intracellular degradation was reported. The CGTase-CDase pathway showed optimal catalytic characteristics at a lower temperature. Based on these observations, we propose that the four enzymes (Tk0977, Tk1770, Tk1774, and Tk2172) participate in the process of starch utilization synergistically with a broad temperature range to provide glucose for cell metabolism (Figure 6).

## Figures and Tables

**Table 1:** Comparison of the biochemical properties of CDase-Tk and those of other CDases from archaea.

	CDase-Tk	CDase-Tc	CDase-Pf					CDase-Tb	CDase-Tp
Homology	100%	59%	56%					53%	52%
aa residues	656	644	645					660	644
Optimal temperature	65	85	90					95	95
Optimal pH	7.5	5.0	4.5					5.5	5.5
Optimal substrate	*β*-CD	*α*-CD	*α*-CD					*β*-CD	*γ*-CD
Substrate preference	CD ≫ PL > SS	CD ≫ MD > SS > PL	CD ≫ MD > SS					CD ≫ MD > SS	CD ≫ MD > PL = SS
Final hydrolysis product	G1	G1, G2	G3, G4					G1, G2	G1, G2
*K*_*m*_ (mg mL^−1^)	3.1	N.D.	*α*-CD	*β*-CD	*γ*-CD	MD	SS	N.D.	N.D.
			2.6	2.2	5.1	62.9	0.5		
*k*_cat_ (s^−1^)	34.6	N.D.	*α*-CD	*β*-CD	*γ*-CD	MD	SS	N.D.	N.D.
			241	196	173	268	67		
*k*_cat_/*K*_*m*_	11.1	N.D.	*α*-CD	*β*-CD	*γ*-CD	MD	SS	N.D.	N.D.
			92.3	90.7	33.8	4.3	128.8		
References	This study	[10]	[12]					[9]	[11]

MD, maltodextrin; CA, cycloamylose; CD, cyclodextrin; PL, pullulan; SS, soluble starch.

The cyclodextrinases are from *T. kodakarensis* KOD1 (CDase-Tk), *Thermococcus* sp. CL1 (CDase-Tc), *P. furiosus* (CDase-Pf), *Thermococcus* sp. B1001 (CDase-Tb), and *Thermofilum pendens* Hrk 5 (CDase-Tp). N.D.:not determined.
